# Lithium-salt anion regulation of ion transport and interfacial stability in PVDF-HFP/PAN/LLZTO composite polymer electrolytes

**DOI:** 10.3389/fchem.2026.1961300

**Published:** 2026-10-21

**Authors:** Yangying Ou, Limin Huang, Lingxiao Lan, Xinghua Liang, Jiajia Dong, Pengzhen Chen, Lei Zhang, Qinglie Mo

**Affiliations:** 1 Guangxi Key Laboratory of Automobile Components and Vehicle Technology, Guangxi University of Science and Technology, Liuzhou, China; 2 School of Mechanical Engineering, Chengdu University, Chengdu, China

**Keywords:** LiODFB, LiTFSI, lithium-ion batteries, performance studies, solid-state electrolytes

## Abstract

**Introduction:**

The growing demand for safer and longer-lasting lithium-ion batteries has highlighted the limitations of conventional liquid electrolytes, particularly their susceptibility to oxidative decomposition and interfacial degradation. Composite polymer electrolytes (CPEs) offer a promising alternative for improving lithium-ion transport and interfacial stability.

**Methods:**

In this study, poly(vinylidene fluoride-co-hexafluoropropylene)/polyacrylonitrile (PVDF-HFP/PAN) was employed as the polymer matrix, with Ta-substituted lithium lanthanum zirconate (LLZTO) incorporated as an inorganic ceramic filler. Lithium bis(trifluoromethanesulfonyl)imide (LiTFSI) and lithium difluoro(oxalato)borate (LiODFB) were separately introduced to investigate the effects of lithium-salt anions on the structural, mechanical, and electrochemical properties of the resulting CPEs.

**Results:**

At a salt loading of 30 wt%, the LiTFSI-based electrolyte (PSI 30%) achieved an ionic conductivity of 6.08 × 10^−4^ S cm^−1^, a tensile strength of approximately 8.5 MPa, and a Li^+^ transference number of 0.58, outperforming the LiODFB-based electrolyte (PFB 30%) in mechanical strength and lithium-ion transport. The LiFePO_4_/PSI 30%/Li cell delivered discharge capacities of 162.61 mAh g^−1^ at 0.1 C and 107.14 mAh g^−1^ at 1 C. After 100 cycles at 0.1 C, the discharge capacity remained above 159 mAh g^−1^, corresponding to approximately 98% capacity retention.

**Discussion:**

These findings suggest that lithium-salt anion selection plays an important role in regulating ionic conductivity, mechanical integrity, and electrochemical performance within PVDF-HFP/PAN/LLZTO composite electrolytes. The LiTFSI-based system exhibits more favorable overall performance, providing useful insights into the design of composite polymer electrolytes for lithium-metal batteries.

## 1 Introduction

As rechargeable Li-based batteries are used under more demanding operating conditions, their electrolyte components must provide higher safety, better interfacial stability, and sustained electrochemical performance (Alawi et al., 2025; Hasan et al., 2025; Sebbagh et al., 2026). Conventional liquid electrolytes can facilitate rapid Li^+^ migration and intimate contact with electrode surfaces; however, their volatility, flammability, and limited thermal resistance still raise serious safety concerns (Xie and Lu, 2025). Under high-temperature, high-voltage, or long-term cycling conditions, liquid electrolytes tend to undergo oxidative degradation, triggering continuous parasitic reactions on electrode surfaces and consequently accelerating impedance buildup and capacity loss (Liu X. et al., 2026; Song et al., 2026). These limitations highlight the need for solid or quasi-solid electrolyte platforms that can maintain Li^+^ transport while improving interfacial durability and battery safety.

Polymer electrolyte systems have attracted considerable attention because their flexible matrices allow facile membrane fabrication, scalable processing, and intimate compatibility with electrode surfaces (Chen et al., 2026; Man et al., 2026). Poly (vinylidene fluoride-co-hexafluoropropylene), abbreviated as PVDF-HFP, was selected to build the polymer framework because its fluorinated backbone provides chemical robustness, oxidation resistance, and thermal endurance. The C-F-containing segments also help maintain membrane integrity under electrochemical operating conditions (Carena et al., 2024; Chen et al., 2024). However, pristine PVDF-HFP electrolytes usually suffer from limited chain mobility and incomplete lithium salt dissociation, which lead to poor room-temperature ion transport and restrict their practical use in lithium batteries (Li et al., 2023). Polyacrylonitrile (PAN) contains abundant polar nitrile groups (-C≡N), which can interact with Li^+^ and reduce cation-anion association, thus facilitating salt dissociation and generating more transport-active Li^+^ species (Yu et al., 2023; Liu et al., 2024; Zhang Y. et al., 2026). Meanwhile, the rigid PAN backbone can strengthen the matrix framework, thereby improving membrane toughness and dimensional stability (Yang et al., 2025). Therefore, incorporating PAN into the PVDF-HFP matrix can optimize Li^+^ migration pathways while preserving the membrane’s flexibility and oxidative tolerance.

Inorganic oxide fillers are commonly introduced into polymer matrices to regulate Li^+^ migration behavior and reinforce interfacial compatibility, leading to the development of composite polymer electrolytes (CPEs) (Fu et al., 2024). Li_6.4_La_3_Zr_1.4_Ta_0.6_O_12_ (LLZTO), as a Ta-doped garnet oxide filler, provides favorable Li^+^ conduction characteristics, electrochemical tolerance, and chemical robustness for polymer electrolyte modification (Wang et al., 2023; Lee et al., 2024; Meng et al., 2026). The incorporation of LLZTO into the PVDF-HFP/PAN framework can weaken regular chain packing and generate more disordered regions, thereby providing more favorable pathways for Li^+^ migration (Wang Y. et al., 2023). In addition, surface active sites of LLZTO may coordinate with functional groups in the polymer chains and immobilize salt-derived anions, which favors Li^+^ release and improves the efficiency of Li^+^ migration (Wang et al., 2022). Furthermore, LLZTO serves as a ceramic reinforcement that strengthens the membrane against thermal deformation and mechanical failure. Meanwhile, the polymer/LLZTO interfacial regions provide additional channels for Li^+^ migration, helping to establish a more continuous ion-conduction framework (Tong et al., 2021; Gan et al., 2023).

Li^+^ mobility and electrode/electrolyte interfacial reactions in composite electrolyte membranes are strongly governed by the anion structure and coordination strength of the selected lithium salt (Wei et al., 2025; Rahman et al., 2026). Lithium bis(trifluoromethanesulfonyl)imide (LiTFSI) contains a TFSI- anion with extensive charge delocalization, which favors salt dissociation and promotes Li^+^ conduction in polymer matrices. In addition, LiTFSI can participate in constructing an inorganic-component-enriched interphase on electrode surfaces, improving interfacial compatibility. Nevertheless, under high-voltage operation, LiTFSI may induce aluminum current collector corrosion (Li et al., 2024; Wang et al., 2025; Perodeau et al., 2026). Yadav et al. investigated a PVDF-HFP-based electrolyte incorporating LiTFSI and LLZTO (Yadav et al., 2023). The membrane reached a Li^+^ conductivity of 1.04 × 10^−4^ S cm^-1^ at 30 °C, while the associated LiFePO_4_/CSPE/Li battery delivered 131 mAh g^-1^ under 0.1 °C at 60 °C. A LiTFSI-modified cationic covalent organic framework, referred to as COF-LiTFSI, was developed as a functional organic additive for polymer electrolytes, enabling more efficient salt ionization and Li^+^ migration and thus improving the overall ionic conduction. Jeong et al. (2026) introduced LiTFSI into Li_6_PS_5_Cl as a healing additive. This modification enhanced the critical current density through suppressed electron leakage within the electrolyte matrix, while facilitating the development of a LiF-enriched interfacial layer with improved mechanical and chemical stability. Lithium difluoro (oxalato)borate (LiODFB) possesses strong interphase-forming capability and can generate a protective interfacial layer composed of B-O species, LiF, and oxalate-derived compounds, which helps mitigate parasitic reactions and enhance interface durability (Tao S.-D. et al., 2021; Gao et al., 2024; Qin et al., 2024). Tao S. et al. (2021) homogeneously incorporated coral-like cubic LLZO particles and LiODFB into the PVDF-HFP framework, creating connected Li^+^ migration channels and improving compatibility with the lithium-metal anode. He et al. (2025) stabilized the Li/solid electrolyte interface and mitigated mechanical degradation by incorporating LiODFB into the electrolyte as a tailored additive. This interphase contains an organic-inorganic mixed exterior and an inorganic-dominated interior, which can suppress electron leakage through the electrolyte and buffer mechanical damage during Li plating/stripping, thereby maintaining stable interfacial contact. Lu et al. proposed and validated the concept of surface functional zoning in SPEs, resolving the issue of performance trade-offs in traditional SPEs and providing new insights into the structural design of high-performance solid-state electrolytes (Lu et al., 2025). They synthesized a locally high-concentration polymer electrolyte (LHCPE), regulated the solvation structures of lithium ions and anions, and promoted anion-derived, LiF-rich interfacial decomposition (Lu et al., 2024). Wei et al. investigated the effect of lithium salt anions on electrolyte performance; the synergistic effect of the -SO_2_F and -SO_2_CF_3_ groups enabled the PGFT-20 electrolyte to form a highly stable SEI film, thereby enhancing interfacial stability with lithium metal (Wei, Qiu, Wang, Gu and Li, 2025). Zhang et al. found that by enhancing the binding energy between LLZO and the polymer, FEC can adsorb onto and wet the LLZO surface, leading to the formation of lithium salt aggregates at the LLZO - PVDF-HFP interface, which increases the number of lithium ion channels and results in high ionic conductivity (Zhang Q. et al., 2026).

Recent studies have demonstrated that not only the chemical identity of lithium salts but also their local coordination environment and spatial distribution can strongly regulate ion transport and interphase formation in polymer electrolytes. Localized-high-concentration, surface-ion-aggregated, and salt-segregated electrolyte architectures have been developed to create Li^+^-rich conduction domains and robust LiF-rich interphases, resulting in improved high-rate capability and lithium-metal compatibility. Nevertheless, most previous studies have focused on a single lithium-salt chemistry or specifically engineered salt-distribution strategy. A direct comparison of different anion chemistries within an identical PVDF-HFP/PAN/garnet composite framework remains relatively limited.

In this work, LiTFSI and LiODFB were systematically compared over an identical salt-loading range of 10–50 wt% in the same PVDF-HFP/PAN/LLZTO host. This controlled comparison enables the influence of anion chemistry to be correlated with polymer coordination, ionic conductivity, mechanical behavior, Li^+^ transference, electrochemical stability, interfacial resistance evolution, and full-cell performance, thereby distinguishing the effect of lithium-salt chemistry from variations in the polymer and ceramic framework.

### 1.1 Materials and chemicals

All reagents were used as received. Poly (vinylidene fluoride) (PVDF, Mn = 600,000) and poly (vinylidene fluoride-co-hexafluoropropylene) (PVDF-HFP, Mn = 400,000) were supplied by Arkema (olombes, France). Polyacrylonitrile (PAN, Mw = 5,000), ithium bis(trifluoromethanesulfonyl)imide (LiTFSI, 99%), lithium difluoro (oxalato)borate (LiODFB, 99%), and N-methyl-2-pyrrolidone (NMP, ≥99%) were obtained from Shanghai Macklin Biochemical Co., Ltd. (China). Ta-doped lithium lanthanum zirconate (Li_6.4_La_3_Zr_1.4_Ta_0.6_O_12_, LLZTO, D_50_ = 0.5 μm) was sourced from Shenzhen Kejing Zhida Technology Co., Ltd. (Guangdong, China). Super P conductive carbon black (≥99.5%) was acquired from Kelude Chemical Technology (Shanghai) Co., Ltd. (China), whereas lithium iron phosphate (LiFePO_4_, LFP, ≥99.5%) was provided by Dongguan Kelude New Energy Technology Co., Ltd. (Guangzhou, China). N,N-dimethylformamide (DMF, 99.8%) was purchased from Aladdin Reagent Co., Ltd. (Shanghai, China).

### 1.2 Preparation of CPEs

As illustrated in Figure 1, for PSI and PFB electrolyte films, a PVDF-HFP/PAN blend with a 7:3 weight ratio was dissolved in DMF at 45 °C under magnetic stirring, yielding a uniform polymer solution. LiTFSI or LiODFB was introduced at salt loadings of 10–50 wt% with respect to the total PVDF-HFP/PAN content. Subsequently, 10 wt% LLZTO powder based on the polymer blend was introduced, followed by another 4 h of stirring until a homogeneous suspension was formed. The resulting slurry was cast with a doctor blade using a 200 μm coating gap, followed by drying at 80 °C for 3 h under reduced pressure. After solvent evaporation, dried membranes were peeled off and cut into 19 mm circular discs. Before cell fabrication, these membranes were stored in an argon glove box for 24 h, where both H_2_O and O_2_ contents were controlled below 0.01 ppm. The samples containing LiTFSI were labeled PSI 10%, PSI 20%, PSI 30%, PSI 40%, and PSI 50%, whereas those containing LiODFB were labeled PFB 10%, PFB 20%, PFB 30%, PFB 40%, and PFB 50%.

**FIGURE 1:**
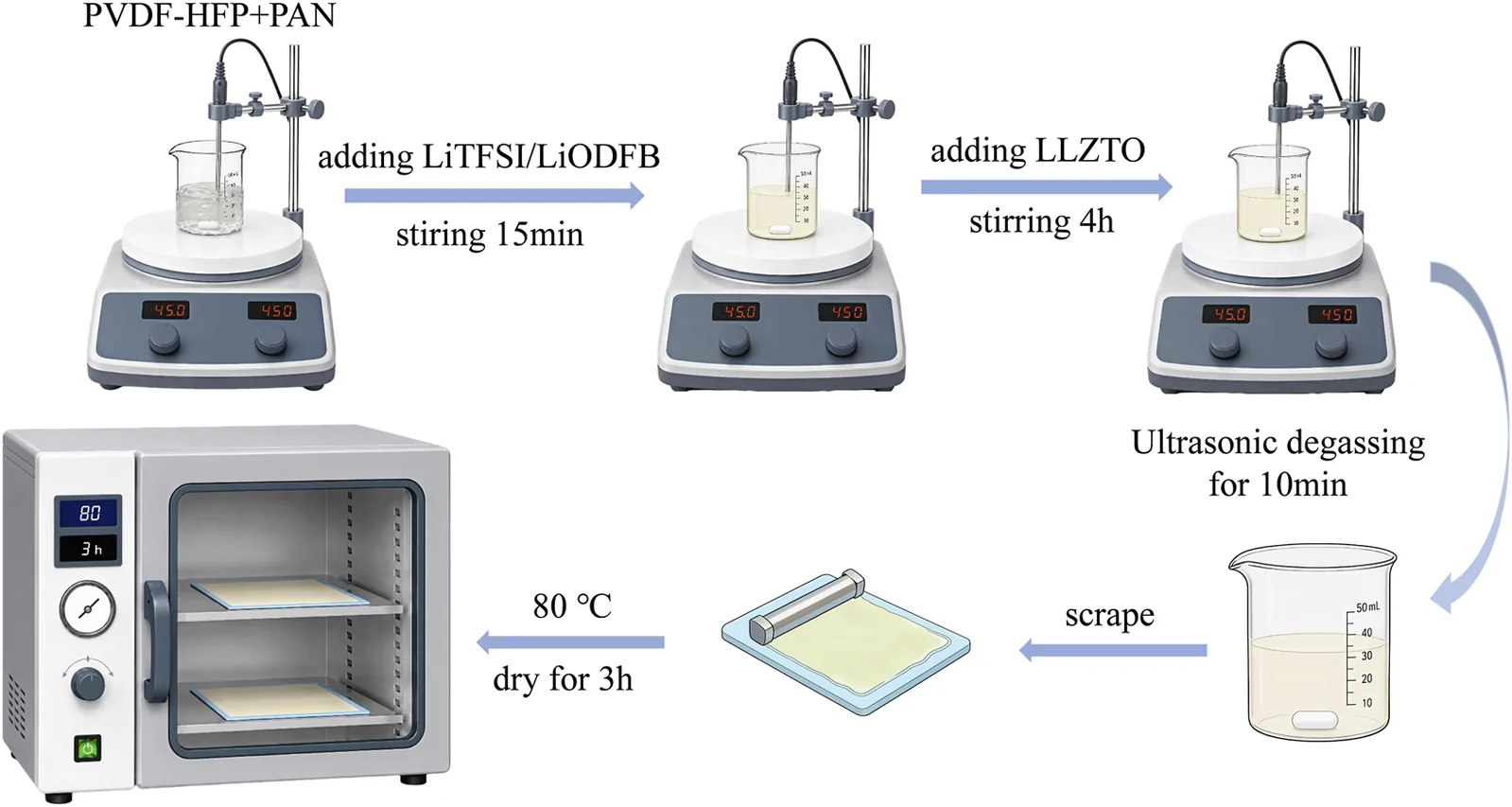
Preparation process of PSI and PFB electrolyte membranes.

### 1.3 Material characterization

The crystalline features of the CPEs membranes were identified by X-ray diffraction using a DX-2700 diffractometer (Dandong, China) equipped with Cu Kα radiation. Diffraction data were recorded over 2θ = 10°–90° at 0.1° min^-1^, with the operating voltage and current fixed at 40 kV and 30 mA, respectively. Surface and fractured-section morphologies were characterized with a field-emission scanning electron microscope (SEM, ZEISS Sigma 36, Germany). For cross-sectional imaging, membrane samples were cryo-fractured in liquid nitrogen and sputter-coated with a thin Au layer. Tensile behavior was evaluated on an electronic universal testing machine (WDW-5, Tenson, Jinan, China) using rectangular strips of 1 × 6 mm. Thermal decomposition behavior was investigated by thermogravimetric analysis (TGA, Netzsch F3 Tarsus, Bayern, Germany) from 30 °C to 900 °C under N_2_ atmosphere at a heating rate of 10 °C min^-1^. Chemical bonding information was obtained from Fourier transform infrared spectroscopy (FTIR, Spectrum 100, PerkinElmer, USA) over 500–4,000 cm^-1^. Surface elemental states and bonding configurations were further analyzed by X-ray photoelectron spectroscopy (XPS, Thermo Scientific K-Alpha, Thermo Fisher Scientific).

### 1.4 Electrochemical properties

Electrochemical impedance spectroscopy (EIS) was used to investigate Li^+^ migration behavior, interface-related resistance, and charge-transfer characteristics in the assembled cells (Ma et al., 2022; Wang P. et al., 2022; Feng et al., 2024). Impedance data were recorded over a frequency window of 10^−1^–10^6^ Hz under a 10 mV AC perturbation. For ion-conduction evaluation, each CPEs film was placed between two mirror-polished steel blocking electrodes to fabricate an SS/CPEs/SS cell. The electrolyte conductivity was then determined from the measured bulk resistance using the following equation:

$$\sigma =\frac{L}{RS}$$ (1)

Here, *σ* is the ionic conductivity expressed in S cm^-1^, *L* denotes the film thickness in cm, *R* represents the bulk resistance derived from the impedance plot in Ω, and *S* refers to the effective electrode/electrolyte contact surface in cm^2^.

Linear sweep voltammetry (LSV) tests were performed with Li/CPEs/SS cells to evaluate membrane voltage stability, and the potential range was set from 2.0 to 6.0 V at a scan rate of 5 mV s^-1^.

Li/CPEs/Li symmetric cells were fabricated in CR2025 coin-cell casings to evaluate the interfacial compatibility between the CPEs membranes and Li metal. The Li^+^ transport number (*t*_*Li*_^*+*^) was obtained through chronoamperometric polarization combined with impedance measurements. A constant DC bias of 10 mV was imposed on the Li/CPEs/Li cells for 4,000 s. Impedance responses were recorded prior to and following polarization over 10^−1^–10^6^ Hz under a 10 mV AC perturbation. The Li^+^ transference number (*t*_*Li*_^*+*^) was determined using the following relation:

$$t_{{Li}^{+}}=\frac{I_{S}\left(\Delta V‐R_{0}I_{0}\right)}{I_{0}\left(\Delta V‐R_{S}I_{S}\right)}$$ (2)

Here, *I*_*0*_ and *I*_*S*_ represent the polarization current at the beginning and at the steady state, expressed in mA; *R*_*0*_ and *R*_*S*_ correspond to the interfacial resistance values measured prior to and following polarization, expressed in Ω; *ΔV* is the applied DC bias, fixed at 10 mV.

LiFePO_4_/CPEs/Li cells were fabricated to investigate the electrochemical performance of these membranes. The cells were first subjected to impedance measurements under open-circuit conditions, and cyclic voltammetry (CV) curves were subsequently collected at 0.2 mV s^-1^.

Rate capability and prolonged cycling performance were tested using a Neware testing system (Guangdong, China). LiFePO_4_ (LFP) was used as the cathode, whereas Li metal foil functioned as the anode. The cathode slurry was formulated by blending LiFePO_4_, PVDF binder, and a conductive carbon additive in NMP at a mass ratio of 8:1:1. After homogenization, the cathode slurry was applied onto Al foil through blade coating, followed by vacuum drying for 36 h. After drying, the cathode films were punched into 14 mm round electrodes for subsequent cell fabrication. These coin cells were cycled over a voltage window of 2.8–4.0 V.

### 1.5 Assembling batteries

For cell fabrication, LiFePO_4_ (LFP) electrodes were employed as the cathodes, while Li metal foil was used as the anode. Before coin-cell construction, the LFP electrodes, Li foils, stainless-steel spacers, and CPEs membranes were dried at 60 °C for 12 h. The resulting CR2025 coin cells were allowed to stand for 12 h before electrochemical measurements.

## 2 Experimental results and analysis

X-ray diffraction (XRD) was employed to characterize the phase features and structural variation of electrolyte membranes with varied lithium-salt loadings. Figure 2a,b display the diffraction profiles of electrolyte films with varied salt loadings. PVDF-HFP presents two main signals at approximately 18° and 20°, while PAN gives a characteristic signal near 16°, suggesting partial crystallinity in the polymer framework. No apparent displacement of the LLZTO-related reflections was observed in the composite electrolyte films, confirming that the garnet-type phase of LLZTO was preserved after membrane fabrication. This result demonstrates the good structural stability of LLZTO in the composite electrolyte membranes (Wang et al., 2026). Upon increasing the lithium-salt loading from 10 to 30 wt%, the diffraction peaks related to the polymer phase in both PSI and PFB gradually weakened and broadened, suggesting that the incorporated salts disturbed PVDF-HFP/PAN chain arrangement and increased structural disorder in the polymer matrix (Zhang et al., 2023). Among these samples, PSI 30% and PFB 30% showed weakened polymer crystalline peaks while retaining the LLZTO crystal phase, indicating that 30 wt% lithium salt provided a suitable balance between increasing the amorphous fraction and preserving the integrity of the electrolyte framework. Further increasing the salt loading to 40 and 50 wt% may lead to localized salt enrichment or phase heterogeneity, which restricts continuous Li^+^ migration within the electrolyte (Zhang et al., 2024).

**FIGURE 2:**
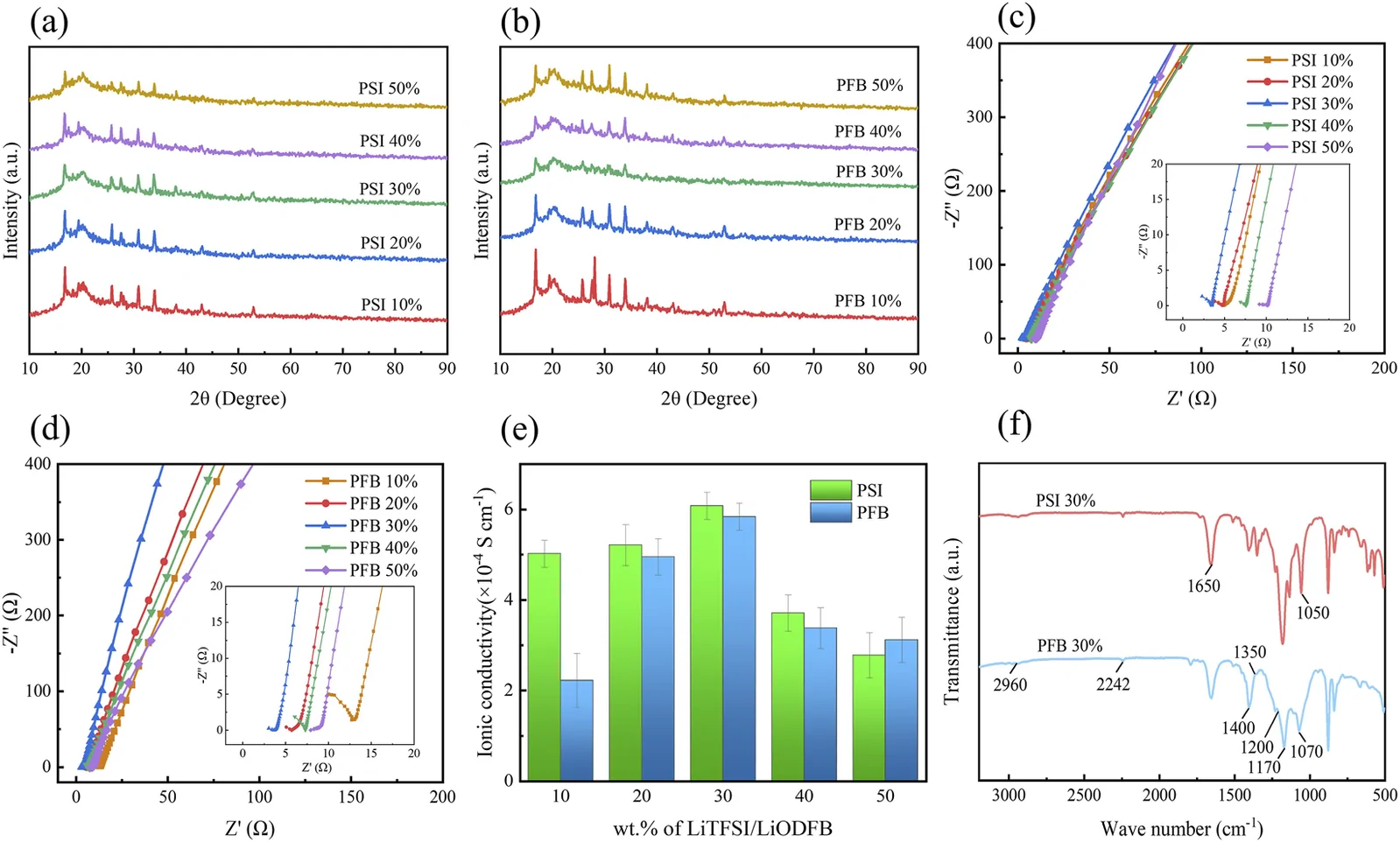
**(a, b)** Diffraction results for CPEs membranes of different formulations; **(c, d)** impedance responses of symmetric stainless-steel/CPEs/stainless-steel cells under ambient conditions, with selected regions magnified in the insets; **(e)** ionic conductivities of CPEs membranes with different compositions, with error bars representing standard deviations; **(f)** FTIR spectra of PSI 30% and PFB 30% electrolytes.

The impedance responses of PSI and PFB electrolyte films are compared in Figures 2c,d while the corresponding resistance and ionic conductivity values are summarized in Tables 1, 2. With LiTFSI content rising across 10–50 wt%, the corresponding resistance values were 4.919, 4.746, 3.482, 7.604, and 10.164 Ω. Under identical LiODFB loadings, the PFB films gave resistance values of 12.719, 5.699, 3.625, 7.313, and 7.924 Ω. With the salt dosage increasing from 10 to 30 wt%, both PSI and PFB showed a continuous reduction in resistance. This behavior indicates that suitable salt incorporation enhances Li^+^ availability and improves chain mobility in the polymer phase, thereby promoting ion conduction. Among these samples, PSI 30% and PFB 30% exhibited the lowest resistance values. At higher salt dosages of 40 and 50 wt%, the resistance rose again, implying that excess salt tends to generate ion-pair or cluster species, suppress polymer-chain mobility, and impede Li^+^ migration (Yang and Wu, 2022). The ionic conductivity results shown in Figure 2e further confirm this trend. The best ion-conducting performance was obtained at 30 wt% salt, giving conductivities of 6.08 × 10^−4^ S cm^-1^ for PSI and 5.84 × 10^−4^ S cm^-1^ for PFB. Considering both the diffraction results and Li^+^ conduction behavior, 30 wt% was identified as the most suitable salt loading for the PSI and PFB electrolyte systems.

**TABLE 1:** Ionic conductivity of PSI electrolytes.

LiTFSI content (wt%)	Resistance (Ω)	Ionic conductivity (×10^−4^ S cm^-1^)
10	4.919	5.02
20	4.746	5.21
30	3.482	6.08
40	7.604	3.71
50	10.164	2.78

**TABLE 2:** Ionic conductivity of PFB electrolytes.

LiODFB content (wt%)	Resistance (Ω)	Ionic conductivity (×10^−4^ S cm^-1^)
10	12.719	2.22
20	5.699	4.95
30	3.625	5.84
40	7.313	3.38
50	7.924	3.12

It should be noted that DMF was used as the solvent during the preparation of the composite membranes. Although complete removal of DMF is difficult due to its high boiling point and strong interaction with Li^+^, the membranes were vacuum-dried at 80 °C for 3 h to remove excess solvent. Previous studies have demonstrated that trace residual DMF can remain confined within PVDF-based polymer matrices after thermal treatment due to its strong coordination with Li^+^ and can participate in Li^+^ solvation, thereby influencing ion transport behavior (Yang et al., 2024; Zhao et al., 2025) The relatively high ionic conductivity of PSI 30% and PFB 30% electrolytes may be partially related to the confined trace DMF molecules, which can facilitate Li^+^ solvation and ion migration within the polymer matrix (Carena et al., 2024). However, the enhanced ionic conductivity mainly originates from the synergistic effects of Li salt dissociation, polymer segmental motion, and LLZTO-assisted ion transport pathways (Fu et al., 2022).

Figure 2f presents the FTIR profiles of PSI 30% and PFB 30%. The main vibration bands associated with PVDF-HFP and PAN are preserved in both membranes. The peak appearing near 2960 cm^-1^ originates from -CH_2_- stretching of the polymer matrix, whereas the feature at about 1400 cm^-1^ is associated with -CH_2_- deformation vibration, suggesting that Li salts and LLZTO were incorporated without destroying the polymer chain structure. Moreover, the feature observed at approximately 2242 cm^-1^ corresponds to nitrile-group stretching vibration in PAN. The persistence and minor shift of the -C≡N signal reveal coordination between Li^+^ and nitrile groups, resulting in stronger local interactions within the polymer network and improved membrane integrity (Liu et al., 2022). For PSI 30%, the TFSI^−^-related absorption signals, mainly arising from -SO_2_- and S-N-S vibrations, appeared between 1350 and 1200 cm^-1^ and at approximately 1070 cm^-1^. The band broadening in PSI 30% suggests that LiTFSI may interact with the polymer matrix through coordination and ion-dipole effects. For PFB 30%, enhanced absorption features near 1650 cm^-1^ and within 1000–1100 cm^-1^ correspond to C=O vibration and B-O vibration associated with the ODFB^−^ anion. The observed peak shifts further indicate that LiODFB was successfully incorporated into the electrolyte system.

Figure 3 shows the surface and cross-sectional SEM images of the PSI 30% and PFB 30% electrolytes, together with their corresponding EDS elemental maps. As shown in Figures 3a,b, the surface of the PSI 30% electrolyte membrane is relatively continuous, with no obvious through-cracks or severe phase separation. The C, O, and F elements are uniformly distributed within the selected regions, indicating good compatibility between LiTFSI and the PVDF-HFP/PAN matrix as well as relatively homogeneous dispersion of the electrolyte components. The PFB 30% electrolyte exhibits a rougher surface morphology, as shown in Figures 3e,f, with numerous pores, a broad pore size distribution, and localized particle agglomeration. In addition, the relatively poor uniformity of the C, O, and F elemental distributions suggests that the LiODFB-containing components are less uniformly dispersed in the electrolyte membrane. Figure 3c, d show the cross-sectional SEM images and corresponding EDS elemental maps. The results further indicate that the PSI 30% electrolyte possesses a relatively dense and continuous internal structure, with C, O, and F elements distributed uniformly along the membrane thickness direction, which is beneficial for constructing continuous Li^+^ transport channels. In contrast, the cross-section of the PFB 30% electrolyte shows more obvious voids and a relatively loose structure (Figures 3g,h). Local enrichment or uneven distribution of C, O, and F elements can be observed in certain regions, which may disrupt ion transport pathways and increase the electrolyte impedance.

**FIGURE 3:**
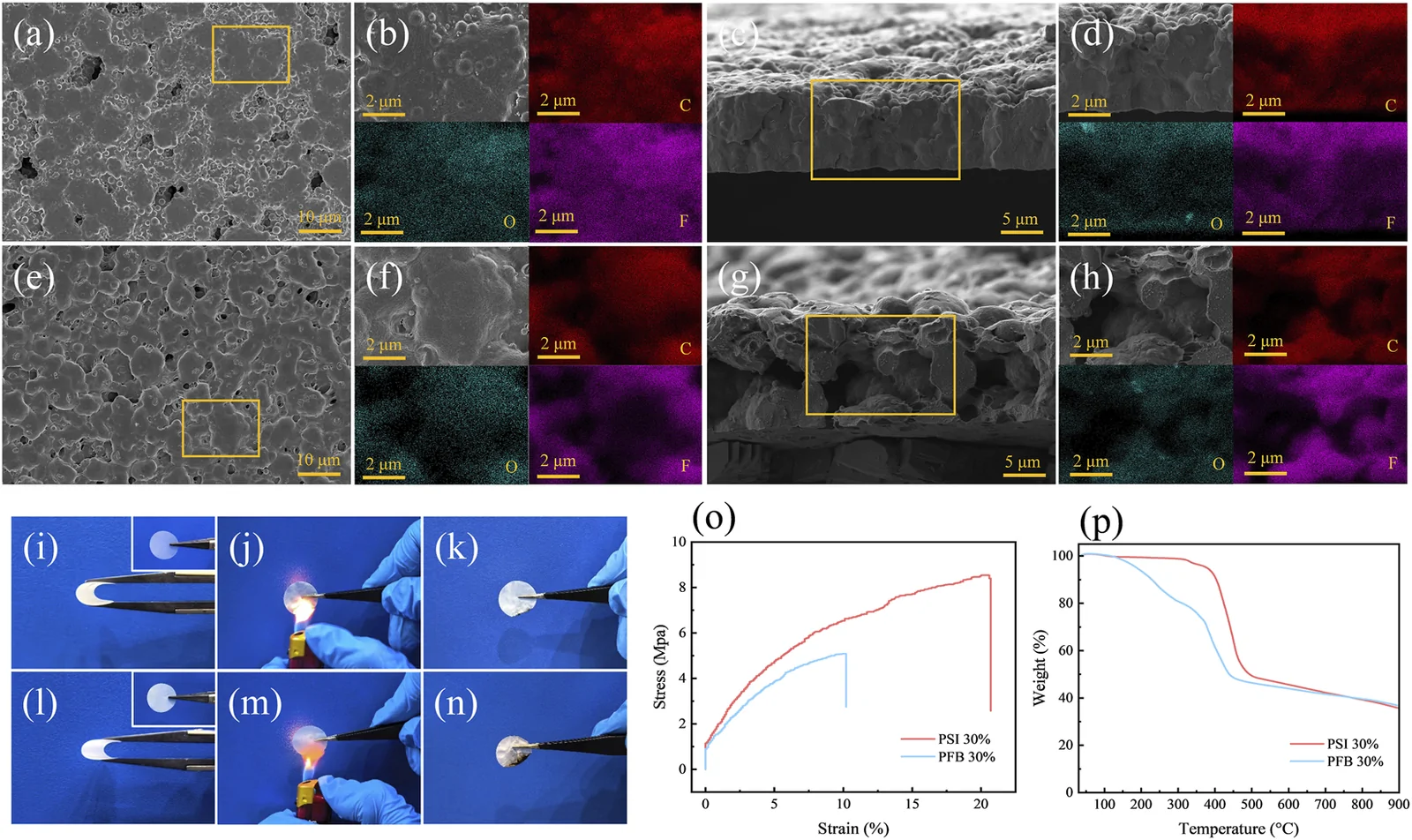
Morphological characterization and EDS mapping results for the PSI 30% membrane: **(a, b)** top-view morphology and **(c, d)** fractured-section morphology; corresponding characterization of the PFB 30% membrane: **(e, f)** top-view morphology and **(g, h)** fractured-section morphology; optical photographs of PSI 30%: **(i)** original and bent states, **(j)** under flame exposure, and **(k)** after flame withdrawal; optical photographs of PFB 30%: **(l)** original and bent states, **(m)** under flame exposure, and **(n)** after flame withdrawal; **(o)** tensile stress-strain profiles and **(p)** thermogravimetric curves of PSI 30% and PFB 30%.

As shown in Figures 3i,l, both electrolyte membranes formed complete disc-shaped self-supporting membranes with milky-white surfaces, and no obvious macroscopic cracks or fragmentation were observed. When folded, both electrolytes maintained a continuous structure without apparent fracture, demonstrating good flexibility and mechanical integrity. As shown in Figures 3m,n, the PFB 30% electrolyte exhibited noticeable wrinkling, shrinkage, and localized morphological damage after removal from the flame source, indicating its relatively poor thermal stability. In contrast, Figure 3j, k show that the PSI 30% electrolyte maintained its circular disc shape relatively well after flame exposure, with only localized carbonization and slight shrinkage, demonstrating certain flame-retardant behavior.

Different lithium salts lead to distinct mechanical properties of the electrolytes. As shown in Figure 3o, the PSI 30% membrane delivers a maximum tensile stress of about 8.5 MPa together with a strain at break of approximately 20%. In contrast, the PFB 30% membrane reaches a peak stress of nearly 5.0 MPa and breaks at a strain of around 10%. Because TFSI^−^ interacts only weakly with Li^+^, LiTFSI favors a more homogeneous distribution of ionic species and increases the mobility of the polymeric framework, which contributes to improved membrane flexibility and fracture resistance. By comparison, the pronounced coordination involving ODFB^−^ may hinder local chain dynamics, thereby increasing membrane rigidity while compromising its deformability (He et al., 2024). Taken together, the PSI 30% membrane combines superior load-bearing capability with enhanced compliance, helping preserve membrane continuity throughout battery fabrication and prolonged electrochemical operation.

Thermal durability of the electrolyte membranes was examined by thermogravimetric analysis (TGA), with the corresponding curves presented in Figure 3p (Li et al., 2021). Both PSI 30% and PFB 30% exhibited negligible weight loss before 150 °C, demonstrating good thermal stability under typical lithium-battery working conditions. Upon heating, the PFB 30% electrolyte membrane showed evident weight loss at a lower temperature, implying an earlier onset of thermal degradation. This behavior is mainly associated with the relatively low thermal stability of the oxalato group in LiODFB. The decomposition of LiODFB at relatively low temperatures may accelerate the degradation of the polymer matrix. By comparison, PSI 30% underwent its main weight-loss stage between 300 °C and 500 °C, mainly arising from degradation of the PVDF-HFP/PAN framework together with partial decomposition of the lithium salt. LiTFSI possesses higher thermal stability, and its -SO_2_-N-SO_2_- structure helps suppress the degradation of polymer chains (Tang et al., 2024). Above 700 °C, the residual masses of the two samples tended to converge, mainly because of the presence of the thermally stable inorganic LLZTO filler. These results indicate that the PSI 30% electrolyte exhibits better structural retention during thermal decomposition and superior thermal stability.

Figures 4a–d presents the high-resolution XPS profiles of PSI 30% and PFB 30%. For PSI 30%, the C 1s spectrum was resolved into multiple contributions corresponding to C-C, C-N, C=O, and -CF_3_ groups. These peaks originate from the PVDF-HFP/PAN polymer backbone, carbon species associated with the nitrile groups in PAN, surface oxygen-containing carbon species, and fluorinated groups in PVDF-HFP and LiTFSI, respectively. The F 1s signals associated with -CF_3_ provide further evidence for fluorinated segments in the polymer framework and -CF_3_ species originating from LiTFSI. In the N 1s spectrum, the -N(SO_2_CF_3_)_2_- related signal, along with the S=O contribution in O 1s, confirms that LiTFSI-derived imide and sulfonyl groups are incorporated into the composite membrane. For the PFB 30% electrolyte, in addition to polymer-related peaks such as C-C, C-N, and-CF_3_ species in the C 1s spectrum, a distinct C=O component is also observed, which primarily originates from the oxalato-borate structure of LiODFB. In the F 1s spectrum, the -CF_3_ related signal mainly originates from PVDF-HFP, while the B-F signal is associated with the difluoro (oxalato)borate structure of LiODFB. In PFB 30%, the N 1s spectrum primarily reflects nitrile groups from PAN, and its fitted components are assigned to uncoordinated -C≡N and Li^+^-coordinated -C≡N species. The distinct B-O-C contribution in the O 1s spectrum confirms that LiODFB was effectively incorporated into the electrolyte membrane (Tao S. et al., 2021). Overall, both lithium salts were successfully incorporated into the PVDF-HFP/PAN/LLZTO composite electrolyte, forming distinct local chemical environments containing fluorine, nitrogen, and oxygen. These chemical environments are beneficial for regulating lithium salt dissociation, Li^+^ coordination, and Li^+^ transport behavior.

**FIGURE 4:**
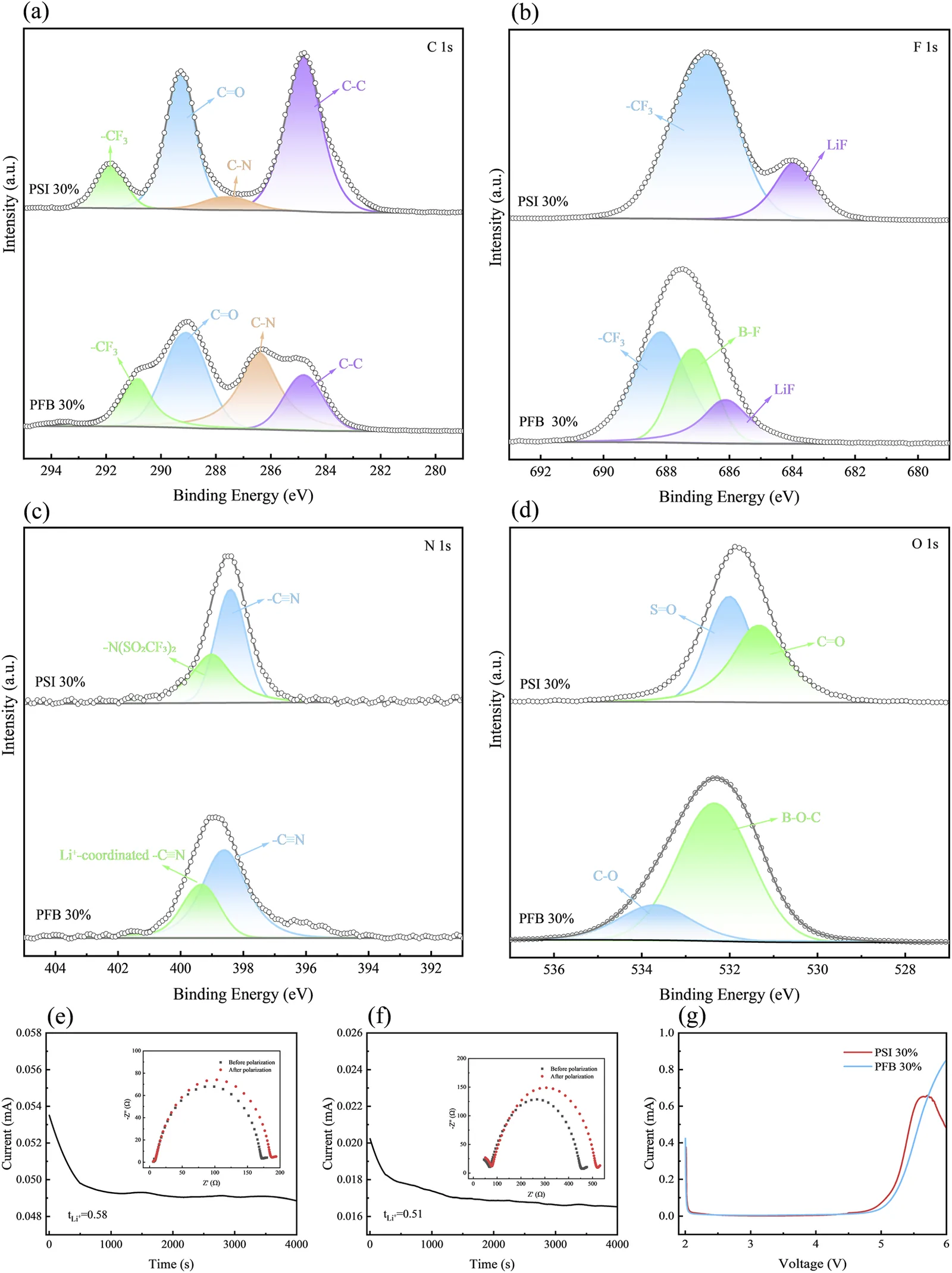
Core-level XPS spectra for PSI 30% and PFB 30%, including C 1s **(a)**, F 1s **(b)**, N 1s **(c)**, and O 1s **(d)**; **(e, f)** chronoamperometric polarization responses of Li/PSI 30%/Li and Li/PFB 30%/Li symmetric cells under a 10 mV bias; the insets show the fitted circuit schemes and impedance responses collected before and after polarization; **(g)** LSV profiles of PSI 30% and PFB 30% electrolytes.

The Li^+^ transport number describes the fraction of total ion conduction carried by Li^+^ in an electrolyte (Du et al., 2019). A higher Li^+^ transference number is favorable for reducing polarization, because it enables more homogeneous Li^+^ redistribution across the electrode/electrolyte boundary, alleviates diffusion-related polarization, and enhances long-term cell durability (Liu H et al., 2026). Figures 4e,f display the chronoamperometric polarization responses of PSI 30% and PFB 30%, together with their impedance profiles recorded prior to and following polarization. At the beginning of polarization, both electrolytes delivered a relatively high current, followed by continuous attenuation toward a stable plateau, suggesting that anion motion was limited and Li^+^ became the main charge carrier. As shown in the inset EIS spectra, the PSI 30% electrolyte exhibited only slight changes in interfacial impedance before and after polarization, demonstrating good interfacial stability. Compared with PSI 30%, PFB 30% exhibited a larger impedance increase after polarization, suggesting more severe polarization. Based on Equation 2, the *t*_*Li*_^*+*^ value of PSI 30% was determined as 0.58, which was higher than that of PFB 30% (0.51). This result indicates that Li^+^ plays a larger role in charge conduction within PSI 30%, which helps mitigate concentration polarization and enhance Li^+^ migration dynamics. The enhanced Li^+^ transference behavior of PSI 30% mainly originates from the relatively weak association of TFSI^−^ with lithium ions, which facilitates salt dissociation and promotes more efficient Li^+^ transport. In contrast, the stronger interaction between ODFB^−^ and Li^+^ in the PFB 30% electrolyte restricts Li^+^ transport to some extent, resulting in a lower Li^+^ transference number.

Linear sweep voltammetry (LSV) was employed to evaluate the electrochemical stability range of PSI 30% and PFB 30%, and the corresponding curves are presented in Figure 4g. Before 4.5 V, both PSI 30% and PFB 30% showed nearly negligible current, demonstrating stable electrochemical behavior under typical battery working voltages. With further increasing potential, the current rose sharply, implying the beginning of oxidative degradation. PSI 30% remained electrochemically stable up to about 4.5 V, while PFB 30% extended this limit to nearly 4.6 V, revealing a slightly broader voltage stability range for the PFB 30% membrane. The enhanced voltage tolerance of PFB 30% is mainly related to LiODFB-derived B-O and C=O functionalities, which favor the construction of a robust interfacial passivation layer and inhibit electrolyte oxidation under high-voltage conditions. In contrast, although the PSI 30% electrolyte exhibits higher ionic conductivity, its interfacial stability is relatively weaker, leading to oxidative decomposition at a lower voltage (Li et al., 2020).

To systematically assess the electrochemical behavior of the two electrolyte systems, PSI 30% and PFB 30% membranes were separately incorporated into LiFePO_4_/CPEs/Li cells for testing. Figure 5a compares the discharge capacities of the two cells under various C-rate conditions. For the LiFePO_4_/PSI 30%/Li cell, the initial discharge capacities reached 162.61, 158.39, 147.58, and 107.14 mAh g^-1^ when the rate increased stepwise from 0.1 to 1 C. After returning to 0.1 C, the discharge capacity recovered to 148.54 mAh g^-1^. For the LiFePO_4_/PFB 30%/Li cell, the initial discharge capacities were 158.13, 152.28, 123.79, and 82.80 mAh g^-1^ as the C-rate was gradually increased from 0.1 to 1 C. When the C-rate returned to 0.1 C, discharge capacity recovered to 145.84 mAh g^-1^. After returning to 0.1 C, both cells exhibited rapid capacity recovery, confirming their strong rate capability and good electrochemical reversibility.

**FIGURE 5:**
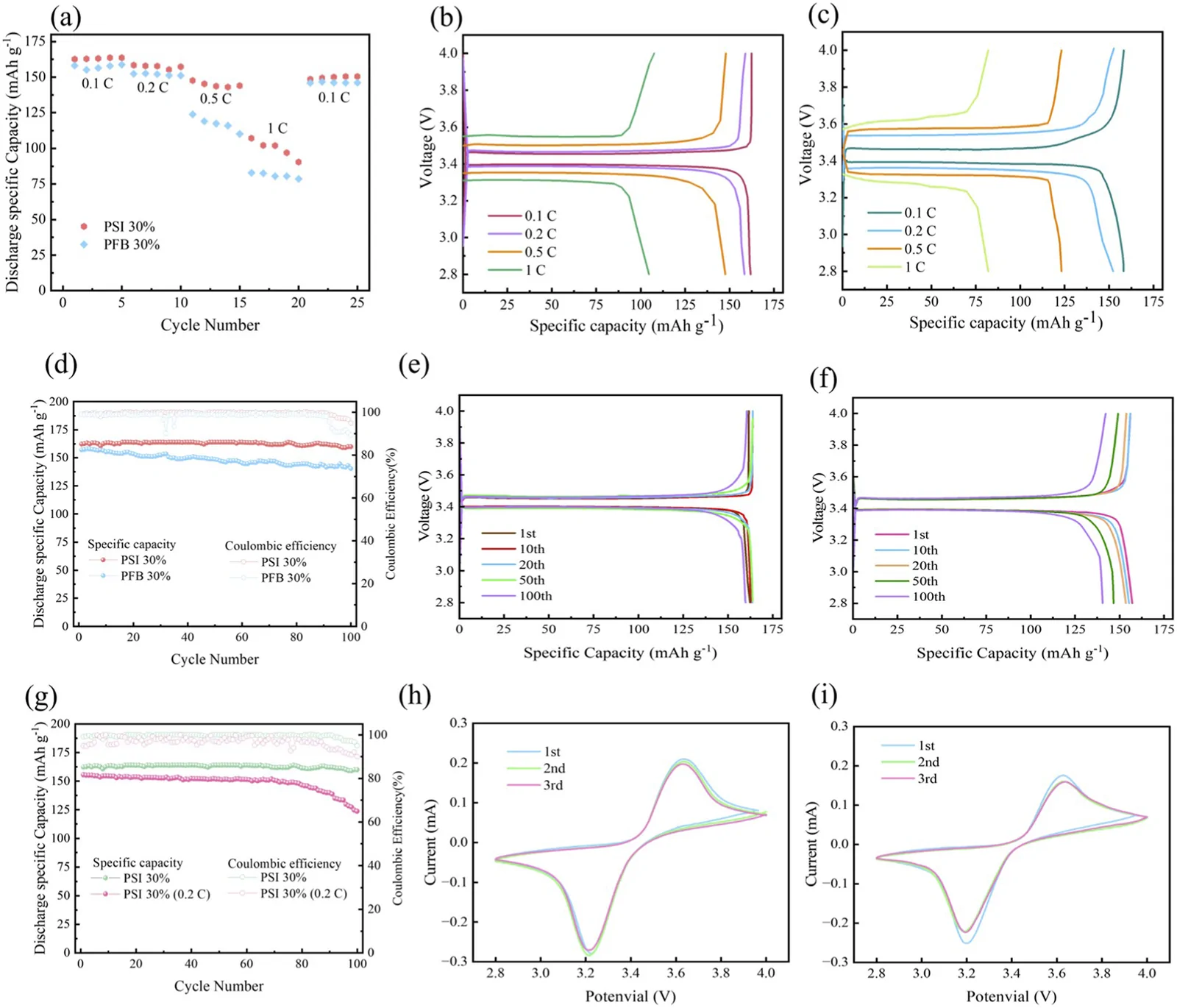
Battery evaluation of LiFePO_4_/PSI 30%/Li and LiFePO_4_/PFB 30%/Li cells: **(a)** rate capability; **(b, c)** galvanostatic charge-discharge curves obtained at 0.1, 0.2, 0.5, and 1 C; **(d)** cycling stability; **(e, f)** representative voltage profiles recorded under 0.1 C after cycles 1, 10, 20, 50, and 100; **(g)** LiFePO_4_/PSI 30%/Li at 0.1 C and 0.2 C cycling stability; **(h, i)** CV curves collected at a scan rate of 0.2 mV s^-1^.

Figures 5b,c display the charge-discharge voltage profiles of the LiFePO_4_/CPEs/Li batteries measured at different C rates. When tested at 0.1 C, the LiFePO_4_/PSI 30%/Li cell showed well-defined plateaus, with the charging plateau near 3.45 V and the discharging plateau around 3.40 V. With increasing C-rate, cell polarization became more pronounced, suggesting higher internal resistance under fast charge-discharge conditions. Nevertheless, both cells maintained distinct and stable charge-discharge plateaus within the voltage range of approximately 3.3–3.6 V. The experimental results indicate that the PSI 30% membrane exhibits better rate performance than the PFB 30% membrane, particularly at high C-rates, where it maintains a higher specific discharge capacity. This suggests that LiTFSI incorporation helps construct interconnected pathways for Li^+^ migration within the composite electrolyte membrane (Hou et al., 2024).

Cycling durability and capacity retention for the two cells were evaluated at ambient temperature at 0.1 C over a voltage range of 2.8–4.0 V (Carter et al., 2025). Figure 5d summarizes the cycling capacities of the two cells, whereas Figures 5e,f present the charge-discharge profiles of PSI 30% and PFB 30% based cells recorded after selected cycles, including cycles 1, 10, 20, 50, and 100.

After 100 cycles, the PSI 30%-based cell still delivered more than 159 mAh g^-1^, indicating good cycling durability despite slight capacity decay during extended operation. In contrast, the PFB 30% based cell exhibited inferior cycling stability. The PFB 30%-based cell provided 157.08 mAh g^-1^ during the initial cycle, whereas the value decreased to 140.45 mAh g^-1^ after 100 cycles. Figure 5e reveals that the PSI 30% based cell maintained highly overlapping voltage profiles throughout cycling, together with steady charging/discharging plateaus and negligible polarization variation, confirming excellent interfacial stability. By comparison, the voltage profiles of the PFB 30% based cell became increasingly separated as cycling proceeded. The discharge plateau moved to lower voltage, whereas the charge plateau shifted to higher voltage, suggesting a continuous increase in polarization. Figure 5g shows the cycling performance and coulombic efficiency of a battery assembled with a 30% PSI electrolyte at 0.1 C and 0.2 C rates. At a 0.2 C rate, the battery initial discharge specific capacity was approximately 155 mAh g^-1^. Capacity decay was relatively slow in the early stages, but gradually accelerated after approximately 70 cycles; after the 100th cycle, the capacity was approximately 123 mAh g^-1^, corresponding to a capacity retention rate of about 80%. As the cycling rate increases, higher current densities exacerbate concentration polarization and electrochemical polarization at the electrode-electrolyte interface, which may lead to a gradual increase in interfacial impedance during long-term cycling, thereby causing capacity loss in the later stages. LiTFSI can favor salt ionization and facilitate more efficient Li^+^ migration. The TFSI^−^ anion possesses a highly delocalized charge structure, which effectively weakens its electrostatic interaction with Li^+^, thereby generating more mobile Li^+^ species and promoting ionic conduction.

As summarized in Table 3, the two CPEs exhibit different performance characteristics despite having the same PVDF-HFP/PAN matrix and LLZTO filler. PSI 30% delivers a higher room-temperature ionic conductivity of 6.08 × 10^−4^ S cm^-1^ and a higher Li^+^ transference number of 0.58 than PFB 30% (5.84 × 10^−4^ S cm^-1^ and 0.51, respectively), indicating more favorable Li^+^ transport. Although PFB 30% shows a slightly wider electrochemical stability window, PSI 30% exhibits better full-cell cycling performance. After 100 cycles at 0.1 C, the LFP/PSI 30%/Li cell maintains a discharge capacity of 159 mAh g^-1^ with a capacity retention of 98%, whereas the LFP/PFB 30%/Li cell delivers 140.45 mAh g^-1^ with a retention of 89.4%. These results demonstrate that lithium-salt selection significantly affects the balance between ion transport, electrochemical stability, and cycling performance, with PSI 30% exhibiting the better overall performance.

**TABLE 3:** Summary of the key properties and full-cell performance of the developed CPEs.

Polymer matrix	Filler	Lithium salt type	σ (S cm^−1^)	t_Li_^+^	LSV	Cell configuration	Discharge capacity at the 100th cycle (mAh g^-1^)	Capacity retention after 100 cycles at 0.1 C (%)
PVDF-HFP/PAN	10%LLZTO	30% LiTFSI	6.08 × 10^−4^	0.58	4.5 V	LFP/PSI 30%/Li	159	98
PVDF-HFP/PAN	10%LLZTO	30% LiTFSI	5.84 × 10^−4^	0.51	4.6 V	LFP/PFB 30%/Li	140.45	89.4

The cyclic voltammetry (CV) curves of the PSI 30% and PFB 30% cells are shown in Figures 5h,i. For the PSI 30% cell, the anodic and cathodic peaks appear at approximately 3.62 and 3.22 V, respectively. For the PFB 30% cell, the anodic and cathodic signals are located at 3.62 and 3.19 V, producing a larger potential separation between the two redox peaks. In addition, the PSI 30% cell exhibits larger oxidation and reduction peak areas than the PFB 30% cell, indicating more favorable redox activity. The PSI 30% cell shows a stronger current response and a narrower redox peak spacing, suggesting faster redox kinetics and reduced polarization. Therefore, the PSI 30% electrolyte contributes to better electrochemical reversibility and improved reaction kinetics in the LiFePO_4_/CPEs/Li cell.

To further highlight the advantages of the CPEs developed in this work, a comparison with previously reported PVDF-HFP-based composite polymer electrolytes containing different fillers and lithium salts is summarized in Table 4. Compared with the reported systems, PSI 30% exhibits higher tensile strength and capacity retention while maintaining relatively high ionic conductivity and Li^+^ transference number. The direct comparison with PFB 30% further indicates that TFSI^−^ favors Li^+^ transport and cycling stability, whereas ODFB^−^ provides a slightly wider electrochemical stability window. These results confirm that lithium-salt anion regulation is an effective strategy for balancing the mechanical properties, ion transport, and full-cell performance of PVDF-HFP-based CPEs.

**TABLE 4:** Comparison of key properties and cell performance of PVDF-HFP-based CPEs.

Polymer matrix	Filler	Lithium salt type	Tensile strength	σ (S cm^−1^)	t_Li_^+^	LSV	Cell Configuration	Loop condition	Capacity retention rate (%)	References
PVDF-HFP	Coral-like LLZO	LiODFB	-	1.65 × 10^−4^	-	-	FePO_4_/CPE/Li	500 cycles at 0.5 C	83.5	Tao S. et al. (2021)
PVDF-HFP/PAN	PDA@LLZTO	LiTFSI	6.92 MPa	4.0 × 10^−4^	-	4.8 V	LiFePO_4_/PPPL-10/Li	400 cycles at 1 C and 0 °C	78.8	Wang Y. et al. (2023)
PVDF-HFP	LATP	LiTFSI	2.27 MPa	5.9 × 10^−4^	0.48	4.7 V	LiFePO_4_/PVHLL-1/Li	1200 cycles at 8 C and RT	82.8	Lu et al. (2024)
PVDF-HFP	LATP	LiTFSI with FEC additive	6.3 MPa	7.8 × 10^−4^	0.69	-	LiFePO_4_/F-PVDF/Li	20,000 cycles at 1.12 A g^-1^	85	Han et al. (2025)
PVDF-HFP	LLZO	LiTFSI with FEC additive	3.75 MPa	7.4 × 10^−4^	0.32	-	LiFePO_4_/PHLLF/Li	640 cycles at 8 C and RT	92.4	Zhang Q. et al. (2026)
PVDF-HFP/PAN	LLZTO	30 wt% LiTFSI	8.5 MPa	6.08 × 10^−4^	0.58	4.5 V	LiFePO_4_/PSI 30%/Li	100 cycles at 0.1 C	98	This work
PVDF-HFP/PAN	LLZTO	30 wt% LiODFB	5.0 MPa	5.84 × 10^−4^	0.51	4.6 V	LiFePO_4_/PFB 30%/Li	100 cycles at 0.1 C	89.4	This work

As shown in Figure 6a, for PSI 30%, the impedance feature related to charge transfer rose from R_10_ = 192.237 Ω after 10 cycles to R_100_ = 716.074 Ω after 100 cycles. As displayed in Figure 6c, PFB 30% showed a larger increase in the corresponding resistance, from 218.750 Ω after 10 cycles to 855.877 Ω after 100 cycles. As the cycle number increased, the impedance of both cells gradually increased, accompanied by a significant increase in interfacial impedance. This indicates the continuous evolution of the electrode/electrolyte interphase during cycling, which leads to enhanced interfacial polarization. Compared with the PFB 30% cell, the PSI 30% cell exhibited a smaller impedance semicircle and a lower impedance growth rate during cycling. Figure 6b and d show the DRT spectra of the PSI 30% and PFB 30% cells, respectively. The PSI 30% cell showed weaker DRT peak intensity and a more centralized peak profile, suggesting reduced interface-related resistance and steadier Li^+^ transport throughout cycling. In contrast, the PFB 30% cell exhibited higher DRT peak intensity, with a marked increase in the medium-to-long relaxation time region, especially within τ = 10^−3^–10^−1^ s, indicating more significant interfacial charge-transfer impedance and diffusion-related polarization. These findings further confirm that PSI 30% helps restrain the progressive buildup of interfacial resistance during cycling, thus contributing to improved cell cycling durability.

**FIGURE 6:**
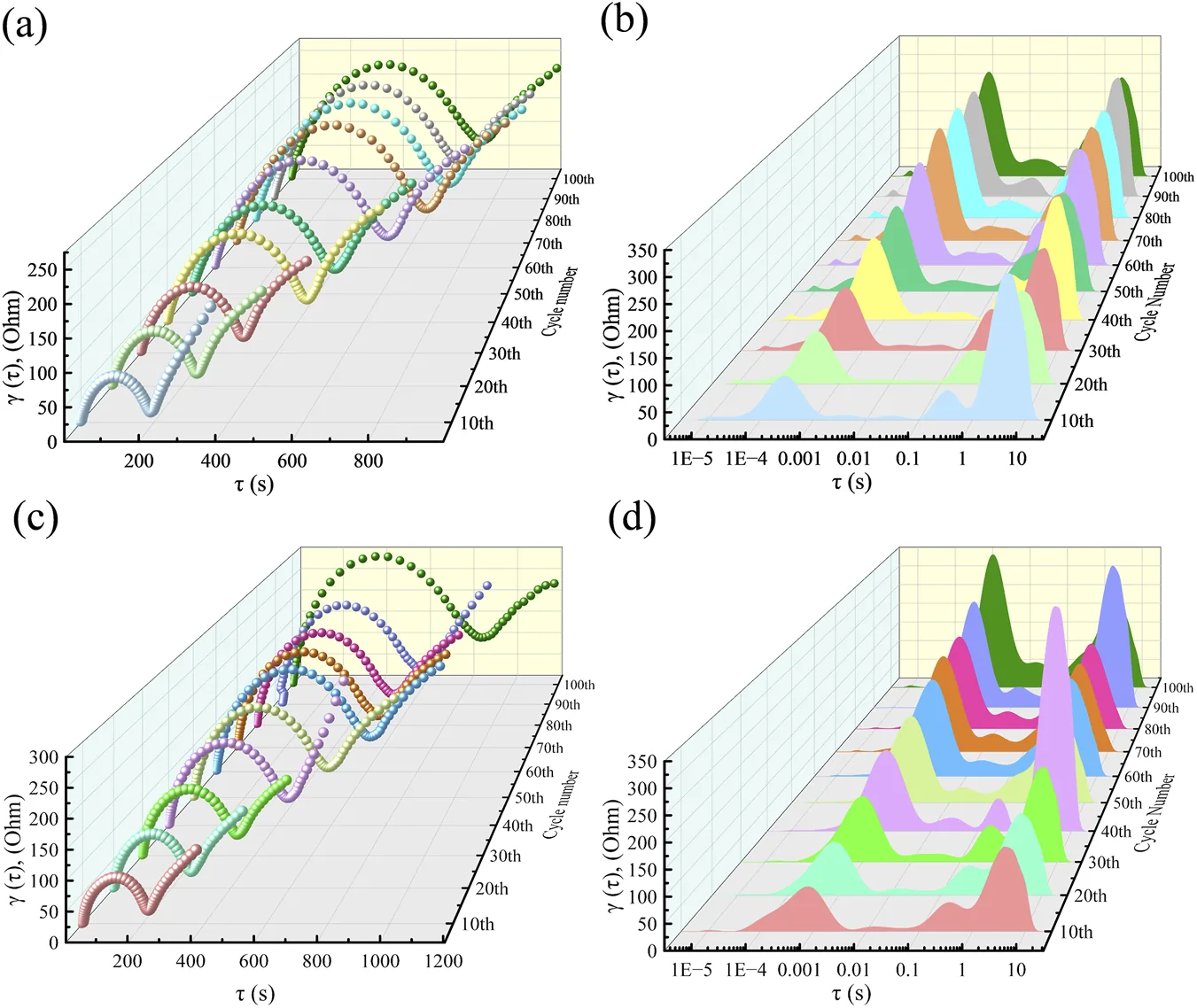
Cycling impedance spectra and DRT analysis of LiFePO_4_/CPEs/Li cells: **(a, b)** evolution of impedance responses and DRT profiles for the PSI 30% cell during cycling; **(c, d)** corresponding impedance responses and DRT profiles for the PFB 30% cell.

To further evaluate the lithium-metal compatibility of the developed composite electrolytes, Li/CPEs/Li symmetric cells were assembled and subjected to galvanostatic Li plating/stripping tests at 0.1 mA cm^-2^. As shown in Figure 7a, both symmetric cells exhibit regular alternating positive and negative voltage plateaus, corresponding to the periodic reversal of the applied current during Li plating and stripping. The enlarged voltage profiles between approximately 174 and 184 h show that the polarization voltages of the PSI 30% and PFB 30% cells are comparable, generally in the range of approximately 25 mV, indicating that the difference between the two electrolytes is not pronounced during this stage. With prolonged cycling, however, the polarization of the Li/PSI 30%/Li cell gradually increases after approximately 180 h and reaches about 40 mV toward 500 h. This gradual increase can be associated with the continuous evolution of the Li/electrolyte interphase during repeated plating/stripping, including changes in the SEI, effective interfacial contact area, charge-transfer kinetics, and concentration polarization (Shi et al., 2018; Fuchs et al., 2023). Galvanostatic polarization is determined not only by interfacial resistance but also by ohmic, SEI-related, charge-transfer/nucleation, and concentration-polarization contributions (Fritzke et al., 2024). Therefore, a moderately higher polarization voltage does not necessarily indicate more severe interfacial degradation. The PFB 30% cell exhibits a somewhat lower apparent polarization during much of the overlapping cycling period, but its voltage amplitude decreases markedly and approaches nearly zero at approximately 390–410 h before the cycling trace terminates. Such an abnormal decrease in polarization may be associated with the development of an incipient soft short or another low-resistance pathway, although additional operando characterization would be required to unambiguously identify the underlying mechanism (Menkin et al., 2024). The post-cycling EIS spectra in Figure 7b further show that the PSI 30% electrolyte exhibits a substantially smaller interfacial impedance than PFB 30%, consistent with the DRT results (Hahn et al., 2020). Taken together, these results indicate that the main advantage of PSI 30% is not a consistently lower plating/stripping overpotential, but rather its ability to sustain reversible Li plating/stripping for 500 h without the pronounced voltage collapse observed for PFB 30%, together with a lower post-cycling interfacial impedance.

**FIGURE 7:**
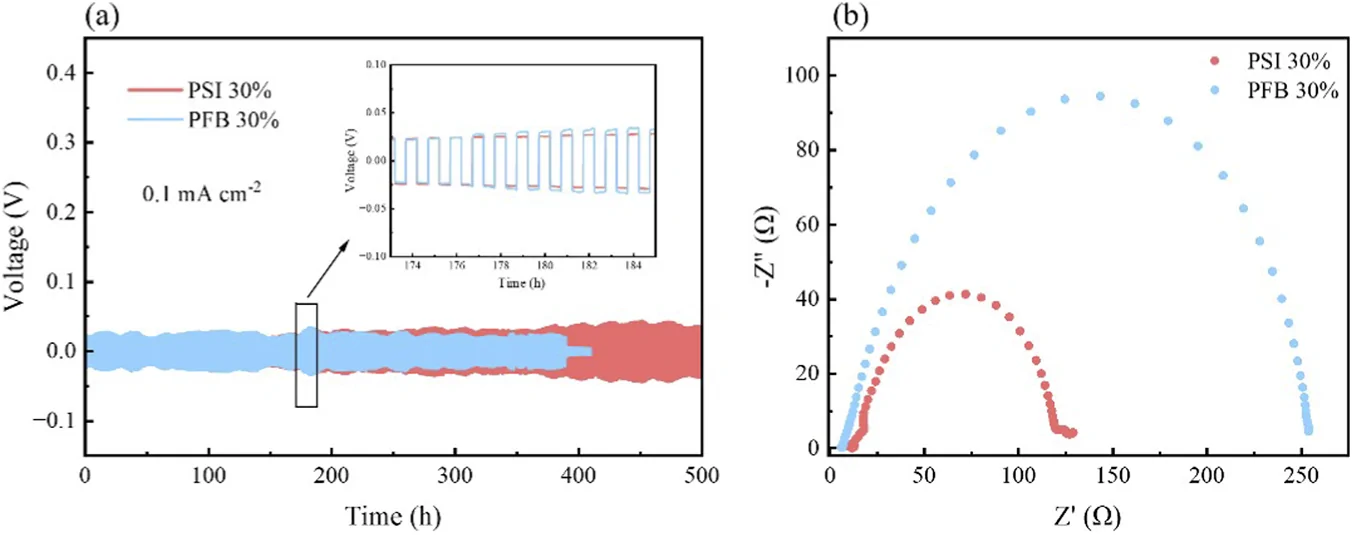
Analysis of lithium deposition/stripping behavior and interface impedance of Li/CPEs/Li symmetric cells: **(a)** Long-term galvanostatic lithium plating/stripping profiles and polarization voltage profiles of Li/PSI 30%/Li and Li/PFB 30%/Li symmetric batteries at a current density of 0.1 mA cm^-2^; **(b)** Corresponding EIS impedance spectra after cycling of the symmetric batteries.

## 3 Conclusion

In the present work, a blended PVDF-HFP/PAN framework was selected as the polymer host, and LLZTO particles were incorporated as the inorganic ceramic phase. To investigate the influence of lithium-salt chemistry and concentration, two membrane series containing LiTFSI or LiODFB at loadings ranging from 10 to 50 wt% were fabricated and evaluated under ambient conditions. For both electrolyte systems, the optimum ionic transport performance was obtained at a salt content of 30 wt%. The ionic conductivities reached 6.08 × 10^−4^ S cm^-1^ in the LiTFSI-based membrane and 5.84 × 10^−4^ S cm^-1^ in the LiODFB-based membrane. At the same salt loading, PSI delivered a tensile strength of 8.5 MPa, along with a Li^+^ transference number of 0.58, both higher than those obtained for PFB, which were 5.0 MPa and 0.51. By comparison, PFB displayed a slightly wider electrochemical stability window, extending to 4.6 V, whereas PSI remained stable up to 4.5 V. The LiFePO_4_/PSI 30%/Li cell delivered 162.61 mAh g^-1^ at 0.1 C and 107.14 mAh g^-1^ at 1 C, whereas the LiFePO_4_/PFB 30%/Li cell achieved 158.13 and 82.80 mAh g^-1^ at the corresponding rates. After 100 cycles, the LiFePO_4_/PSI 30%/Li cell retained a discharge capacity of more than 159 mAh g^-1,^ corresponding to a capacity retention of approximately 98%. These results demonstrate that lithium-salt selection strongly affects ion transport, mechanical integrity, electrochemical stability, and battery performance in PVDF-HFP/PAN/LLZTO composite electrolytes, thereby providing useful guidance for designing safer lithium-based solid-state batteries.

## Data Availability

The original contributions presented in the study are included in the article/supplementary material, further inquiries can be directed to the corresponding authors.

## References

[B1] Alawi A. Saeed A. Sharqawy M. H. Al Janaideh M. (2025). A comprehensive review of thermal management challenges and safety considerations in lithium-ion batteries for electric vehicles. Batteries 11, 275. 10.3390/batteries11070275

[B2] Carena E. Mezzomo L. Vallana N. Ceribelli N. Di Liberto G. Mostoni S. (2024). PVDF-HFP based, quasi-solid nanocomposite electrolytes for lithium metal batteries. Small 20, 2311805. 10.1002/smll.202311805 38409572

[B3] Carter M. Poudel P. Iriyama T. Liaw B. Zhang G. (2025). Effects of low-temperature pre-cycling on high-temperature cycle life of lithium-ion cells. Energy Mater. Devices 3, 9370078. 10.26599/EMD.2025.9370078

[B4] Chen J. Wang H. Bai Y. Pang P. Zheng Z. Xu H. (2024). Dual-MOFs-cage constructed multistage-channel PVDF-HFP quasi-solid electrolytes for lithium metal battery. J. Power Sources 614, 234973. 10.1016/j.jpowsour.2024.234973

[B5] Chen J. Chen H. Armand M. Brunklaus G. Choi J. W. He Y. B. (2026). Toward a unified mechanistic understanding of polymer electrolytes for advanced solid-state batteries. Adv. Mater. 38, e73750. 10.1002/adma.73750 PMC13411047 42318617

[B6] Du Z. Wood Iii D. L. Belharouak I. (2019). Enabling fast charging of high energy density Li-ion cells with high lithium ion transport electrolytes. Electrochem. Commun. 103, 109–113. 10.1016/j.elecom.2019.04.013

[B7] Feng Z. Qiu X. Chen X. Wang H. Guo X. (2024). Interfacial dynamics study of NCM523-based semi-solid-state lithium-ion batteries by electrochemical impedance spectroscopy. ACS Appl. Mater. Interfaces 16, 42995–43005. 10.1021/acsami.4c09122 39092637

[B8] Fritzke J. B. Ellison J. H. Brazel L. Horwitz G. Menkin S. Grey C. P. (2024). Spiers memorial lecture: lithium air batteries–tracking function and failure. Faraday Discuss. 248, 9–28. 10.1039/d3fd00154g 38105743 PMC10823487

[B9] Fu J. Li Z. Zhou X. Guo X. (2022). Ion transport in composite polymer electrolytes. Mater. Adv. 3, 3809–3819. 10.1039/d2ma00215a

[B10] Fu Y. Gu Z. Gan Q. Mai Y.-W. (2024). A review on the ionic conductivity and mechanical properties of composite polymer electrolytes (CPEs) for lithium batteries: insights from the perspective of polymer/filler composites. Mater. Sci. Eng. R. Rep. 160, 100815. 10.1016/j.mser.2024.100815

[B11] Fuchs T. Haslam C. G. Richter F. H. Sakamoto J. Janek J. (2023). Evaluating the use of critical current density tests of symmetric lithium transference cells with solid electrolytes. Adv. Energy Mater. 13, 2302383. 10.1002/aenm.202302383

[B12] Gan H. Sun J. Xia Y. Qiu P. Li L. Zhu W. (2023). PEO/PVDF-HFP/LLZTO composite solid polymer electrolyte for high-performance all-solid-state lithium metal batteries. J. Phys. Chem. C 127, 21015–21021. 10.1021/acs.jpcc.3c06275

[B13] Gao H. Chen Y. Teng T. Yun X. Lu D. Zhou G. (2024). Interface engineering via manipulating solvation chemistry for liquid lithium-ion batteries operated ≥ 100° C. Angew. Chem.Int. Ed. 136, e202410982. 10.1002/anie.202410982 38935427

[B14] Hahn M. Rosenbach D. Krimalowski A. Nazarenus T. Moos R. Thelakkat M. (2020). Investigating solid polymer and ceramic electrolytes for lithium-ion batteries by means of an extended distribution of relaxation Times analysis. Electrochim. Acta 344, 136060. 10.1016/j.electacta.2020.136060

[B15] Han X. Lu J. Zou Q. Wang H. Chen M. Liu T. (2025). Salt-segregated solid polymer electrolytes for high-rate solid-state lithium batteries. Adv. Mater. 37, 2504419. 10.1002/adma.202504419 40159852

[B16] Hasan M. M. Haque R. Jahirul M. I. Rasul M. G. Fattah I. M. R. Hassan N. M. S. (2025). Advancing energy storage: the future trajectory of lithium-ion battery technologies. J. Energy Storage 120, 116511. 10.1016/j.est.2025.116511

[B17] He J.-z. Yan X. Luo L.-f. Qiu H.-d. Peng J.-H. Ma Q.-Q. (2024). Study on synergistic effect and high temperature performance of dual-salts of LiTFSI and LiODFB in LiCoO_2_-based batteries. Solid State Ion. 412, 116584. 10.1016/j.ssi.2024.116584

[B18] He Z. Liu Y. Liu Y. Xiao H. Xie Z. Sheng C. (2025). Hybrid coating on sulfide electrolytes via lithium salts additive for stable all-solid-state lithium metal batteries. ACS Nano 19, 33523–33533. 10.1021/acsnano.5c10768 40936105

[B19] Hou H. Wang Y. Liu X. Yu X. Rong J. Niu Y. (2024). High performance solid state lithium batteries with a continuous homogeneous Li^+^ transmission channels. J. Energy Storage 89, 111737. 10.1016/j.est.2024.111737

[B20] Jeong S. Kim C. Avvaru V. S. Teeter G. Ahn J. Yang G. (2026). Self-healing lithium dendrites through spontaneous passivating layer formation for stable solid-state lithium-metal batteries. ACS Nano 20, 10198–10209. 10.1021/acsnano.6c01373 41849699 PMC13045351

[B21] Lee Y.-G. Hong S. Pan B. Wu X. Dickey E. C. Whitacre J. F. (2024). Improving bulk and interfacial lithium transport in garnet-type solid electrolytes through microstructure optimization for high-performance all-solid-state batteries. ACS Appl. Mater. Interfaces 16, 60340–60347. 10.1021/acsami.4c13891 39465543 PMC11551947

[B22] Li Y. Cheng B. Jiao F. Wu K. (2020). The roles and working mechanism of salt-type additives on the performance of high-voltage lithium-ion batteries. ACS Appl. Mater. Interfaces 12, 16298–16307. 10.1021/acsami.9b22360 32216273

[B23] Li L. Xie M. Zhang Y. Xu Y. Li J. Shan Y. (2021). Thermal safety and performances analysis of gel polymer electrolytes synthesized by *in situ* polymerization for Li-ion battery. J. Solid State Electrochem. 25, 2021–2032. 10.1007/s10008-021-04965-3

[B24] Li H. Feng T. Liang Y. Wu M. (2023). Construction of PMIA@PAN/PVDF-HFP/TiO_2_ coaxial fibrous separator with enhanced mechanical strength and electrolyte affinity for lithium-ion batteries. Chin. Chem. Lett. 34, 108350. 10.1016/j.cclet.2023.108350

[B25] Li Z. Wang L. Huang X. He X. (2024). Lithium bis (trifluoromethanesulfonyl) imide (LiTFSI): a prominent lithium salt in lithium‐ion battery electrolytes-fundamentals, progress, and future perspectives. Adv. Funct. Mater. 34, 2408319. 10.1002/adfm.202408319

[B26] Liu C. Sacci R. L. Sahore R. Veith G. M. Dudney N. J. Chen X. C. (2022). Polyacrylonitrile-based electrolytes: how processing and residual solvent affect ion transport and stability. J. Power Sources 527, 231165. 10.1016/j.jpowsour.2022.231165

[B27] Liu X. Bi Z. Wan Y. Guo X. (2024). Composition regulation of polyacrylonitrile-based polymer electrolytes enabling dual-interfacially stable solid-state lithium batteries. J. Colloid Interface Sci. 665, 582–591. 10.1016/j.jcis.2024.03.166 38552575

[B28] Liu H. Jing H. Chen Y. Li J. Xing H. Liu Y. (2026). Integrating vertical magnetic fields for safe lithium metal batteries with fast lithium-ion kinetics. J. Power Sources 666, 239080. 10.1016/j.jpowsour.2025.239080

[B29] Liu X. Jamadon N. H. Yu Y. Zheng L. Tang R. (2026). Electrolyte design and interface engineering for high-voltage solid-state lithium batteries. Front. Chem. 14, 1840199. 10.3389/fchem.2026.1840199 42211403 PMC13212338

[B30] Lu J. Sheng B. Chen M. Xu M. Zhang Y. Zhao S. (2024). Localized high concentration polymer electrolyte enabling room temperature solid-state lithium metal batteries with stable LiF-rich interphases. Energy Storage Mater 71, 103570. 10.1016/j.ensm.2024.103570

[B31] Lu J. Sheng B. Fang Q. Chen H. Li C. Wang B. (2025). Spatially surface ion-aggregated solid polymer electrolyte with robust mechanical properties for lithium metal batteries. eScience 6, 100462. 10.1016/j.esci.2025.100462

[B32] Ma Y. Sun Q. Wang S. Zhou Y. Song D. Zhang H. (2022). Li salt initiated *in-situ* polymerized solid polymer electrolyte: new insights via *in-situ* electrochemical impedance spectroscopy. Chem. Eng. J. 429, 132483. 10.1016/j.cej.2021.132483

[B33] Man B. Zhao Y. Fu B. Zeng Y. Chen Y. Liu Q. (2026). Multifunctional design of solid polymer electrolytes: paving the way for high-performance all-solid-state batteries. Carbon Energy 8, e70188. 10.1002/cey2.70188

[B34] Meng M. Fu R. Ma K. Yang J. Ding Y. Lu R. (2026). Synergistic cyclodextrin-LiTFSI coating on LLZTO toward high ionic conductivity PVA-based composite electrolytes. ACS Appl. Mater. Interfaces 18, 12991–13003. 10.1021/acsami.5c21921 41705482

[B35] Menkin S. Fritzke J. B. Larner R. de Leeuw C. Choi Y. Gunnarsdóttir A. B. (2024). Insights into soft short circuit-based degradation of lithium metal batteries. Faraday Discuss. 248, 277–297. 10.1039/d3fd00101f 37870402 PMC10823489

[B36] Perodeau T. Vargas J. A. Dollé M. Laventure A. (2026). Exploring the impact of LiTFSI on solid polymer electrolytes crystallization behavior. Polymer, 352, 129839. 10.1016/j.polymer.2026.129839

[B37] Qin G. Zhang J. Chen H. Li H. Hu J. Chen Q. (2024). Lithium difluoro (oxalate) borate as electrolyte additive to form uniform, stable and LiF-rich solid electrolyte interphase for high performance lithium ion batteries. Surf. Interfaces 48, 104297. 10.1016/j.surfin.2024.104297

[B38] Rahman S. Pereira R. Nedsaengtip J. Kalra V. (2026). In-operando FTIR study to investigate the effect of varying lithium salts on solid electrolyte interface (SEI) evolution in lithium metal batteries. Adv. Sci. 13, e23503. 10.1002/advs.202523503 41837871 PMC13116073

[B39] Sebbagh T. Manfo T. A. Şahin M. E. (2026). Advances in battery technologies for next-generation energy storage systems. Electronics 15, 690. 10.3390/electronics15030690

[B40] Shi F. Pei A. Boyle D. T. Xie J. Yu X. Zhang X. (2018). Lithium metal stripping beneath the solid electrolyte interphase. Proc. Natl. Acad. Sci. U.S.A. 115, 8529–8534. 10.1073/pnas.1806878115 30082382 PMC6112724

[B41] Song L. Qin X. Wu D. Xiao X. Xiao Z. Kuang Y. (2026). Interfacial problems and optimization strategies at the oxide solid-state electrolyte-electrode interface. Ionics 32, 5839–5860. 10.1007/s11581-026-07167-x

[B42] Tang C. Yu L. Jiang Q. Gu R. Zhang Y. Liu Z. (2024). Polymer-based electrolyte for lithium-based high-energy-density and safe energy storages devices: strategy and mechanisms. Renewables 2, 297–340. 10.31635/renewables.024.202400068

[B43] Tao S. Li J. Wang L. Huang W. Zhang M. Chi Z. (2021). Coral-like Li_7_La_3_Zr_2_O_12_-filled PVDF-HFP/LiODFB composite electrolytes for solid-state batteries with excellent cycle performance. ACS Appl. Energy Mater. 4, 11447–11459. 10.1021/acsaem.1c02217

[B44] Tao S.-D. Li J. Hu R. Wang L. Chi Z. Li T. (2021). 3Li_2_S-2MoS_2_ filled composite polymer PVDF-HFP/LiODFB electrolyte with excellent interface performance for lithium metal batteries. Appl. Surf. Sci. 536, 147794. 10.1016/j.apsusc.2020.147794

[B45] Tong R.-A. Chen L. Fan B. Shao G. Liu R. Wang C.-A. (2021). Solvent-free process for blended PVDF-HFP/PEO and LLZTO composite solid electrolytes with enhanced mechanical and electrochemical properties for lithium metal batteries. ACS Appl. Energy Mater 4, 11802–11812. 10.1021/acsaem.1c02566

[B46] Wang Y. Li P. Liu B. Wei X. Fu W. Li H. (2025). Solid polymer electrolytes: ion conduction enhancement and comprehensive frontiers. Mater. Futur. 4, 042103. 10.1088/2752-5724/ae0ce3

[B47] Wang Y. Wu J. Chen W. Jiang Z. J. Jiang Z. (2026). Lithium dendrite growth mechanisms and inhibition strategies in garnet-type solid-state electrolytes: a review. Small 22, e13300. 10.1002/smll.202513300 41574893

[B48] Wang L. Zhong Y. Wen Z. Li C. Zhao J. Ge M. (2022). A strong Lewis acid imparts high ionic conductivity and interfacial stability to polymer composite electrolytes towards all-solid-state Li-metal batteries. Sci. China Mater. 65, 2179–2188. 10.1007/s40843-021-1908-x

[B49] Wang P. Yan D. Wang C. Ding H. Dong H. Wang J. (2022). Study of the formation and evolution of solid electrolyte interface via *in-situ* electrochemical impedance spectroscopy. Appl. Surf. Sci. 596, 153572. 10.1016/j.apsusc.2022.153572

[B50] Wang Q. Wang Y. He X. Ma Y. Zhang D. Li Z. (2023). *In-situ* constructing of dual bifunctional interfacial layers of garnet-type Li_6.4_La_3_Zr_1.4_Ta_0.6_O_12_ solid electrolyte towards long-cycle stability for flexible solid metal lithium batteries. J. Power Sources. 580, 233469. 10.1016/j.jpowsour.2023.233469

[B51] Wang Y. Chen Z. Wu Y. Li Y. Yue Z. Chen M. (2023). PVDF-HFP/PAN/PDA@LLZTO composite solid electrolyte enabling reinforced safety and outstanding low-temperature performance for quasi-solid-state lithium metal batteries. ACS Appl. Mater. Interfaces 15, 21526–21536. 10.1021/acsami.3c02678 37071843

[B52] Wei Y.-M. Qiu A. Wang J. Gu Y. Li J.-F. (2025). Influence of lithium salt anions on the interfacial properties of PEO-Based solid-state electrolytes. Electrochem. Commun. 177, 107979. 10.1016/j.elecom.2025.107979

[B53] Xie J. Lu Y. C. (2025). Designing nonflammable liquid electrolytes for safe Li-ion batteries. Adv. Mater. 37, 2312451. 10.1002/adma.202312451 38688700 PMC11733716

[B54] Yadav P. Hosen M. S. Dammala P. K. Ivanchenko P. Van Mierlo J. Berecibar M. (2023). Development of composite solid polymer electrolyte for solid-state lithium battery: incorporating LLZTO in PVDF-HFP/LiTFSI. Solid State Ion. 399, 116308. 10.1016/j.ssi.2023.116308

[B55] Yang H. Wu N. (2022). Ionic conductivity and ion transport mechanisms of solid-state lithium-ion battery electrolytes: a review. Energy Sci. Eng. 10, 1643–1671. 10.1002/ese3.1163

[B56] Yang W. Liu Y. Sun X. He Z. He P. Zhou H. (2024). Solvation-tailored PVDF-Based solid-state electrolyte for high-voltage lithium metal batteries. Angew. Chem.Int. Ed. 136, e202401428. 10.1002/anie.202401428 38470429

[B57] Yang M. Li L. Wang Z. Li Z. Fu A. Li H. (2025). Preparation of LATP@SI-PAN-PEO composite solid electrolyte and the studies of its electrochemical performance. Colloids Surf. A Physicochem. Eng. Asp. 728, 138759. 10.1016/j.colsurfa.2025.138759

[B58] Yu X. Li M. Wei L. Qiu J. Cao G. Wen Y. (2023). Application of polyacrylonitrile in the electrolytes of lithium metal battery. Prog. Chem. 35, 390–406. 10.7536/pc220913

[B59] Zhang D. Meng X. Hou W. Hu W. Mo J. Yang T. (2023). Solid polymer electrolytes: ion conduction mechanisms and enhancement strategies. Nano Res. Energy 2, e9120050. 10.26599/nre.2023.9120050

[B60] Zhang Y. Zhang L. Zhao Z. Zhang Y. Cui J. Liu C. (2024). Lithiophilic Li-Si alloy-solid electrolyte interface enabled by high-concentration dual salt-reinforced quasi-solid-state electrolyte. J. Energy Chem. 95, 216–230. 10.1016/j.jechem.2024.03.031

[B61] Zhang Q. Ji D. Ding W. Ni Q. Zhu W. Li C. (2026). Localized surface segregation of lithium salts enabling fast lithium-ion channels and robust interphases toward stable solid-state lithium batteries. Chin. Chem. Lett. 37, 113223. 10.1016/j.cclet.2026.113223

[B62] Zhang Y. Ganguly D. Tandon A. Yan T. Guan F. Ghosh S. (2026). Ultrathin and mechanically robust polyacrylonitrile-based solid polymer electrolytes enabling long-cycle-life lithium metal batteries. Adv. Fiber Mater. 8, 1–12. 10.1007/s42765-026-00726-x

[B63] Zhao F. Wu J. Qin C. Jiang Z. J. Chen G. Maiyalagan T. (2025). Research progress on the enhancement and modification of PVDF-Based polymer electrolytes and their applications in solid-state lithium metal batteries. Chem. Asian J. 20, e202401974. 10.1002/asia.202401974 40019312

